# Rigid Finite Element Method in Modeling Composite Steel-Polymer Concrete Machine Tool Frames

**DOI:** 10.3390/ma13143151

**Published:** 2020-07-15

**Authors:** Paweł Dunaj, Krzysztof Marchelek, Stefan Berczyński, Berkay Mizrak

**Affiliations:** 1 Department of Mechanical Engineering and Mechatronics, West Pomeranian University of Technology, 71-310 Szczecin, Poland; krzysztof.marchelek@zut.edu.pl (K.M.); stefan.berczynski@zut.edu.pl (S.B.); 2 Mechanical Engineering Department, Yildiz Technical University, 34349 Beşiktaş/İstanbul, Turkey; info@berkaymizrak.com

**Keywords:** rigid finite element method, composite, steel-polymer concrete, machine tool, multibody system

## Abstract

At the stage of designing a special machine tool, it is necessary to analyze many variants of structural solutions of frames and load-bearing systems and to choose the best solution in terms of dynamic properties, in particular considering its resistance to chatter. For this reason, it is preferred to adopt a low-dimensional calculation model, which allows the user to reduce the necessary calculation time while maintaining a high accuracy. The paper presents the methodology of modeling the natural frequencies, mode shapes, and receptance functions of machine tool steel welded frames filled with strongly heterogenous polymer concrete, using low-dimensional models developed by the rigid finite elements method (RigFEM). In the presented study, a RigFEM model of a simple steel beam filled with polymer concrete and a frame composed of such beams were built. Then, the dynamic properties obtained on the basis of the developed RigFEM models were compared with the experimental results and the 1D and 3D finite element models (FEM) in terms of accuracy and dimensionality. As a result of the experimental verification, the full structural compliance of the RigFEM models (for beam and frame) was obtained, which was manifested by the agreement of the mode shapes. Additionally, experimental verification showed a high accuracy of the RigFEM models, obtaining for the beam model a relative error for natural frequencies of less than 4% and on average 2.2%, and for the frame model at a level not exceeding 11% and on average 5.5%. Comparing the RigFEM and FEM models, it was found that the RigFEM models have a slightly worse accuracy, with a dimensionality significantly reduced by 95% for the beam and 99.8% for the frame.

## 1. Introduction

One of the alternative technologies for manufacturing frames forming machine tool support systems is the technology of welding steel bodies. The basic components of such bodies are hollow steel profiles made of sheet metal or solid rolled profiles. Those profiles can be filled with a specially selected composite material whose task it is to increase the dissipation of vibration. Then, such a body is a hybrid body and it can effectively compete with cast iron body technology.

The use of hybrid body technologies (i.e., those resulting from the combination of different materials in order to exploit their properties synergically) in machine tool load-bearing systems manufactured individually or in small batches can provide specific benefits in terms of a significant reduction in the design time, particularly in terms of the calculations needed to shape their structure and the desired static and dynamic properties [1]. It is particularly important to shape the desired resistance of the designed machine tool frame to chatter [2,3,4,5,6,7]. The problems related to the design and modeling of frames of technological machines include a significant number of issues, which translate into a rich scientific literature. Analyzing the review publications on the currently proposed solutions, a large variety in their design can be noticed [8,9,10,11,12,13,14]. The static and dynamic properties of composite machine tool load-bearing systems are most often assessed using the finite element model (FEM) [15,16,17,18,19,20,21,22].

In [23], the authors present a structural solution of a gantry milling machine tool bed composed of a welded steel frame filled with polymer concrete. This solution was aimed at improving the dynamic properties of the machine tool. In relation to the fact that the required stiffness of the body was ensured by the welded frame and its good damping properties were provided primarily by the filling material, six different structural variants were proposed. The subsequent variants differed in the wall thickness of the steel frame and, consequently, in the volume of the filled internal spaces of the elements forming the frame. To assess the impact of the changes in the wall thickness of the static stiffness, natural frequencies, and mode shapes, the finite element models of the proposed variants were analyzed using the Ansys 6.0 software. The results of the model calculations were compared with the experimental ones, both for the values of the estimated loss factor and the natural frequencies; the relative errors were obtained at the level of up to 20%.

In [24], the authors present a solution for the construction of the column body of the milling machine, consisting of a cast iron frame, concrete, and elastomeric material, aimed at improving the damping properties of the structure. The body design process was supported by the analysis of its FEM model. The experimental verification of the results of the FEM modeling showed discrepancies in the values of natural frequencies at a level not exceeding 2.3% of the relative error.

In [25], the authors discuss a solution consisting of replacing the steel body elements of a table milling machine with composite parts made of aluminum, steel, polymer concrete, and carbon fibre-based composites. The models were built using the ANSYS software. Cubic elements characterized by eight nodes (SOLID45) were used to discretize the aluminum, steel, and polymer concrete parts. A layered version of the mentioned finite element (SOLID46) was used for the discretization of the parts made of the carbon fibre-based composite. A modal analysis of the body constraint in the places of foundation of the actual structure was carried out for the constructed model. By verifying the obtained results of modeling with the experimental data, discrepancies in the values of natural frequencies were obtained at a level of up to 28%.

In [26], the authors present a solution consisting of replacing a cast iron vertical milling center column with a steel-reinforced polymer concrete structure. The purpose of the presented research was to increase the damping capacity of the structure while ensuring the required rigidity. The authors, using FEM, conducted an analysis of the structure deformations for seven body variants differing in their arrangement and reinforcement method. A similar approach is presented in [27], replacing the cast iron base of the milling center with a steel-reinforced epoxy granite structure.

In [28], the authors present an enhancement of the static and dynamic characteristics of a micro-lathe bed by the use of alternate form designs and composite material (epoxy granite and nettle polyester). Using the finite element method, the authors analyze six design variants differing in rib configurations. Based on the performed analysis, the best solution in terms of bending and torsional stiffness was selected. However, the authors of the papers [26,27,28] do not provide the results of the experimental verification of the developed finite element models of the hybrid machine tool body elements.

The prognostic value of the results of the calculations made in the design phase will depend directly on the accuracy of the mapping of the static and dynamic properties of the designed body by the calculation model. Since calculations are usually performed for many considered structural solution variants—i.e., they are repeated many times—it is advisable to adopt a design model that is as simple as possible and as complex as necessary. This can be achieved by the rigid finite element model [29], which, compared to the currently most widely used finite element model, has a much smaller number of degrees of freedom, which can significantly reduce the calculation time [30,31].

According to the latest contribution [32], a strongly heterogenous material, such as the polymer concrete analyzed, can be modeled using a linear-elastic isotropic model of material, if present in a steel-polymer concrete beam. Moreover, it is possible to develop a reliable analytical model of a composite steel-polymer concrete beam using a homogeneous beam model with an equivalent bending stiffness and equivalent mass per unit length of the beam. The presented study takes this a step further and proposes a modeling methodology that deals with the problem of modeling steel beams and complex, spatial, steel-welded frames filled with strongly heterogenous material, while maintaining a low dimensionality of the model. The main novelty of the presented study is establishing the model of the composite, steel-polymer concrete beam using the rigid finite element method. The model is built by aggregating matrices describing the inertia and stiffness of the steel outer and polymer concrete filling. The proposed approach differs from the one presented so far in the literature, where mainly the finite element method is used to model the dynamic properties of complex composite structures; in order to reduce the dimensions of the established models, model order reduction methods are used. The presented paper contains the results of comparative studies to determine whether the rigid finite element method can be effectively used in modeling and calculating the dynamic properties—i.e., the natural frequencies, mode shapes, and frequency response functions—of steel structures filled with a highly heterogenous polymer concrete.

The structure of the article is as follows: in Section 2, the steel-polymer concrete beam and frame concepts are described. Next, a static test to determine the properties of the polymer concrete analyzed and a dynamic test to determine the natural frequencies, mode shapes, and frequency response functions are presented. Then, finite element models of both the structural component (beam) and main frame of the vertical lathe are presented. Finally, in Section 3 rigid finite element models are established and compared with the finite element model and experimental results. Section 4 contains the discussion of the presented results. In Section 5, the main conclusions are presented.

## 2. Materials and Methods

### 2.1. Steel-Polymer Concrete Frame

**Figure 1:**
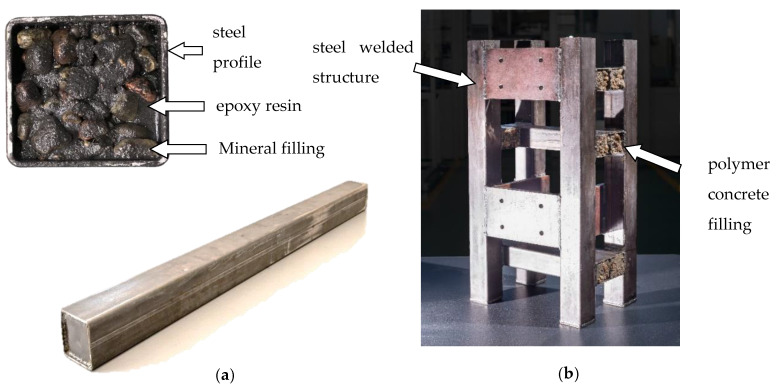
Steel-polymer concrete structure: (**a**) beam, (**b**) steel-polymer concrete frame of vertical lathe.

The frame considered in this paper was made of commercial steel profiles with a square hollow section. The individual profiles (basic structural components) were welded, creating a spatial structure which provides the construction with the required stiffness. After the steel frame was welded, the insides of the profiles were filled with polymer concrete. The use of polymer concrete was intended to increase the damping properties of the welded steel frame. Moreover, filling a steel profile with polymer concrete causes a local increase in the stiffness and inertia of the frame. Therefore, the proper, intentional placement of polymer concrete enables the shaping of the dynamic properties of the machine frame. More precisely, the proper distribution of the polymer concrete causes a change in the structure and parameters of the mass-spring-damping system, which is a model reflection of the machine tool, thus changing its dynamic properties. The basic structural component (beam) and frame analyzed are shown in Figure 1.

The basic structural component under consideration consisted of a steel profile with section dimensions of 50 mm × 50 mm, a wall thickness of 2 mm, and a length of 1000 mm filled with polymer concrete. The frame analyzed consisted of basic structural components and steel plates connected by welded joints. 

The applied polymer concrete consisted of epoxy resin and a mineral filling of various granulations. Considering the grain size as the dividing criteria, the mineral fillings can be grouped into the following categories: (i) ash, (ii) a fine fraction (mainly sand) of grain size 0.25–2 mm, (iii) a medium fraction of grain size 2–10 mm, and (iv) a coarse fraction of grain size 8–16 mm. The coarse (iv) and medium (iii) fractions mainly consisted of irregularly shaped gravel. The mass percentage of the individual components of the polymer concrete applied is shown in Table 1 [32].

### 2.2. Static Tests of Polymer Concrete

To determine the material properties required for the RigFEM modeling—i.e., the Young’s modulus and Poisson ratio—static tests were performed using Instron 8850 (Instron, Norwood, MA, USA). The samples (cuboidal samples of steel—50 mm × 50 mm × 110 mm; polymer concrete—45 mm × 45 mm × 110 mm) were conditioned for 72 h in an air-conditioned laboratory at a temperature of 23 °C and relative humidity of 50%. The detailed procedure was presented in [32]. Table 2 contains the material data determined from the static test and the loss factor determined by the impulse test.

### 2.3. Dynamic Experimental Tests of the Basic Structural Component and Frame

**Figure 2:**
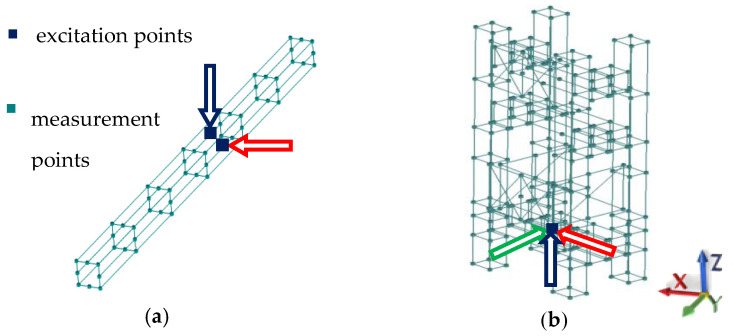
Measurement points’ arrangement for (**a**) beam and (**b**) frame.

Dynamic tests were performed in the form of an impact test using Siemens TestLab—Desktop Advance 2019.1 (Siemens, Munich, Germany) software and Scadas Mobile Vibco (Siemens, Munich, Germany) hardware. Excitation was carried out using a PCB 086C01 (PCB Piezotronics, Depew, New York, NY, USA) modal hammer in two perpendicular axes in the case of the beam analyzed and in three perpendicular axes in the case of the frame analyzed. The response measurement was carried out using three-axis PCB 356A01 ICP (PCB Piezotronics, Depew, New York, NY, USA) accelerometers at 56 points in the case of the beam and 260 points in the case of the frame. Double impacts or the overload of any channel result in the automatic rejection of measurement. The measurement points’ arrangement is shown in Figure 2. The tested beam was suspended on steel cables to approximate free-free boundary conditions, while the frame was founded according to the real operating conditions.

The selected signal acquisition parameters are presented in Table 3.

As a result of the experiment, 112 frequency response functions for the beam and 780 for the frame were determined using the H1 type of FRF estimator. The modal model was estimated using the Polymax algorithm [33] with default values of tolerance on frequency, 1%; damping, 2%; and mode shapes vector, 5%. Before the final interpretation, the models were validated using an modal assurance criterion (MAC) indicator. The detailed procedure for the beam was presented in [32] and for the frame in [34].

**Figure 3:**
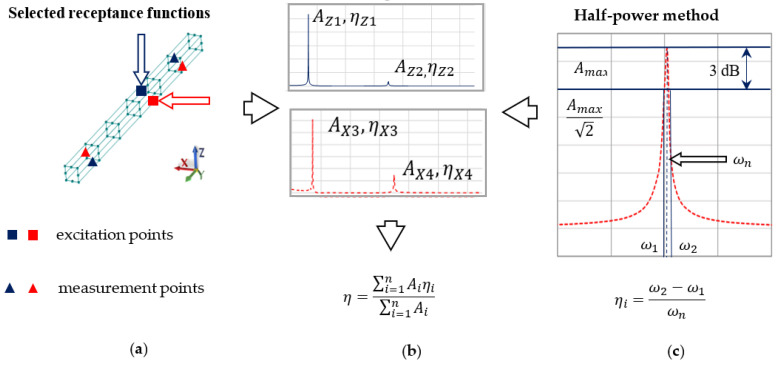
Method of determining the loss factor based on (**a**) selected frequency response functions, (**b**) weighted arithmetic mean, and (**c**) half-power method.

Then, using a half-power method (Figure 3c), the value of the loss factors for steel and steel-polymer concrete beams were determined. First, the frequency response functions between the points indicated in Figure 3a were determined. Those points were selected on the basis of the modal analysis in such a way that they were not in the nodes of the experimental mode shapes. Next, the values of the loss factor for steel and the equivalent loss factor for the composite steel-polymer concrete beam were determined as a weighted arithmetic mean, giving weight to the individual coefficients η corresponding to the resonance amplitude for which they were determined. The idea of the described method is presented in Figure 3b; the values of the loss factors determined are presented in Table 4.

### 2.4. Finite Element Modeling

As indicated in Section 1, in engineering practice, the finite element method (FEM) is most commonly used to assess the dynamic properties of composite structures. Therefore, finite element models of the steel-polymer concrete beam and frame under consideration were built and the corresponding calculations were made.

The beam was modeled according to the method presented in [34], and the steel profile and the polymer concrete filling were meshed using cubic, isoparametric, eight-noded solid elements (CHEXA) and isoparametric, wedge, and solid elements with six nodes (CPENTA). A structural mesh was applied to increase the efficiency of the FEM. The polymer concrete was modeled as a linear-elastic isotropic material (MAT1) [35]. In total, the model of a single beam was composed of 19,600 elements and 21,985 degrees of freedom.

Alternatively, the steel-polymer concrete beam was modeled using one-dimensional beam finite elements (CBAR), formulated on the basis of classical beam theory [35]. The model was built by aggregating mass and stiffness matrixes representing the steel outer and polymer concrete filling. In total, the one-dimensional steel-polymer concrete beam model was composed of 40 elements and 126 degrees of freedom.

**Figure 4:**
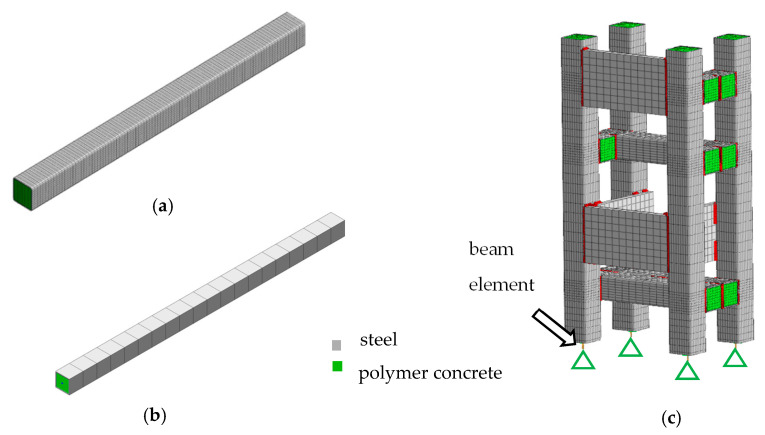
Finite element model (FEM) of a beam discretized using 3D elements (**a**) and 1D elements (**b**) and the model of a frame (**c**).

In the case of the analyzed frame, the model was built according to [34] using the CHEXA and CPENTA elements. In total, the model of the machine tool frame was composed of 60,584 elements and 225,384 degrees of freedom. The support was modeled using a beam element (CBAR) with an equivalent stiffness and damping. The discretization process was performed using Midas NFX 2018 R1 (Midas Information Technology Co. Ltd., Seongnam, Korea) [35,36]. The discretized models are shown in Figure 4.

## 3. Rigid Finite Element Modeling

### 3.1. Rigid Finite Element Model of the Basic Structural Component

In previous research [32], it was proved analytically and experimentally confirmed that it is possible to build a reliable model of a composite steel-polymer concrete beam using an equivalent bending stiffness and an equivalent mass per unit length. Adopting the Hamiltonian developed in [32] and the model assumptions that the contact of the steel profile with the polymer concrete filling occurs on the entire inner surface of the steel profile and the adhesion forces prevent tangential movements within the material contact area, the steel-composite beam was modeled using rigid finite elements characterized by mass and moments of inertia, which were connected to one another using spring-damping elements [37,38].

According to the adopted assumptions, a steel-polymer concrete beam can be modeled as an assembly of steel and polymer concrete parts, determining for them the substitute values of inertia, stiffness, and damping coefficients. Therefore, the matrices describing the inertia, stiffness, and damping of the model are defined as follows Equations (1)–(3):

(1) $$M=M^{st}+M^{pc}$$

(2) $$K=K^{st}+K^{pc}$$

(3) $$C=C^{st}+C^{pc}$$

where M is the inertia matrix of the rigid finite element, $M^{st}$ is the inertia matrix of the steel part, $M^{pc}$ is the inertia matrix of the polymer concrete part, K is the stiffness coefficient matrix describing the spring-damping element, $K^{st}$ is the stiffness matrix describing the steel part, $K^{pc}$ is the stiffness matrix describing the polymer concrete part, C is the damping coefficient matrix describing the spring-damping element, $C^{st}$ is the damping coefficient matrix for the steel part, and $C^{pc}$ is the damping coefficient matrix for the polymer concrete part.

The inertia matrix of a rigid finite element for the beam under consideration is a diagonal matrix whose form is as follows Equations (4)–(6):

(4) $$M=diag\left(M_{1}^{st},M_{2}^{st},\ldots ,M_{6}^{st}\right)+diag\left(M_{1}^{pc},M_{2}^{pc},\ldots ,M_{6}^{pc}\right)$$

whereby:

(5) $$M_{1}^{st}=M_{2}^{st}=M_{3}^{st}=m^{st},\;\;M_{4}^{st}=J_{0}^{st},\;\;M_{5}^{st}=J_{2}^{st},\;\;M_{6}^{st}=M_{5}^{st}$$

(6) $$M_{1}^{pc}=M_{2}^{pc}=M_{3}^{pc}=m^{pc},\;\;M_{4}^{pc}=M_{0}^{pc},\;\;M_{5}^{pc}=J_{2}^{pc},\;\;M_{6}^{pc}=M_{5}^{pc}$$

where m is the mass of the rigid finite element for the polymer concrete filling (*pc*) and the steel coating (*st*), $J_{0}^{st}$ is the main central moment of inertia for the steel profile, $J_{0}^{pc}$ is the main central moment of inertia for the polymer concrete filling, $J_{2}^{st}$ is the inertia for the steel profile relative to the X axis, and $J_{2}^{pc}$ is the moment of inertia for the polymer concrete filling relative to the X axis—Figure 3.

The stiffness K matrix of the spring-damping element is defined as follows Equations (7)–(9):

(7) $$K=diag\left(K_{1}^{st},K_{2}^{st},\ldots ,K_{6}^{st}\right)+diag\left(K_{1}^{pc},K_{2}^{pc},\ldots ,K_{6}^{pc}\right)$$

whereby:

(8) $$K_{1}^{st}=\frac{E^{st}A^{st}}{\Delta l},\;K_{2}^{st}=\frac{G^{st}A^{st}}{\Delta l},\;K_{3}^{st}=K_{2}^{st}\;,\;K_{4}^{st}=\frac{G^{st}J_{0}^{st}}{\Delta l}\;,\;K_{5}^{st}=\frac{E^{st}J_{2}^{st}}{\Delta l},\;K_{6}^{st}=K_{5}^{st}$$

(9) $$K_{1}^{pc}=\frac{E^{pc}A^{pc}}{\Delta l},\;K_{2}^{pc}=\frac{G^{pc}A^{pc}}{\Delta l},\;K_{3}^{pc}=K_{2}^{pc},\;K_{4}^{st}=\frac{G^{pc}J_{0}^{pc}}{\Delta l}\;,\;K_{5}^{pc}=\frac{E^{pc}J_{2}^{pc}}{\Delta l},\;K_{6}^{pc}=K_{5}^{pc}$$

where $E^{st}$ is the Young’s module for steel, $E^{pc}$ is the Young’s module for polymer concrete, $A^{st}$ is the steel profile sectional area, $A^{pc}$ is the polymer concrete filling sectional area, $G^{st}$ is the Kirchhoff’s module for steel, $G^{pc}$ is the Kirchhoff’s module for polymer concrete, and Δl is the length of the rigid element. 

Meanwhile, the damping matrix C of the spring-damping element according to the adopted complex stiffness model [39,40] takes the following form Equation (10):

(10) $$C=i\eta \left(diag\left(K_{1}^{st},K_{2}^{st},\ldots K_{6}^{st}\right)+diag\left(K_{1}^{pc},K_{2}^{pc},\ldots ,K_{6}^{pc}\right)\right)$$

where i—imaginary unit; η—equivalent loss factor for a steel-polymer concrete beam.

**Figure 5:**
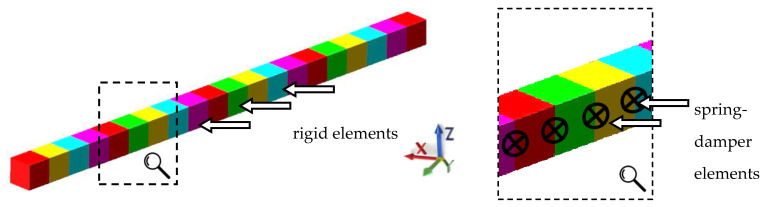
Division of the steel-polymer concrete beam into rigid finite elements.

The beam model using the predefined rigid finite elements was built in MSC Adams 2019 (MSC Software, Newport Beach, California, USA) [41]. The division of the beam under consideration into rigid finite elements and spring-damping elements is shown in Figure 5. The model consisted of 20 rigid finite elements of equal length and 19 spring-damping elements (modeled in MSC Adams environment using 6 × 6 field elements). The model had 120 degrees of freedom. 

### 3.2. Rigid Finite Element Model of the Steel-Polymer Concrete Body

**Figure 6:**
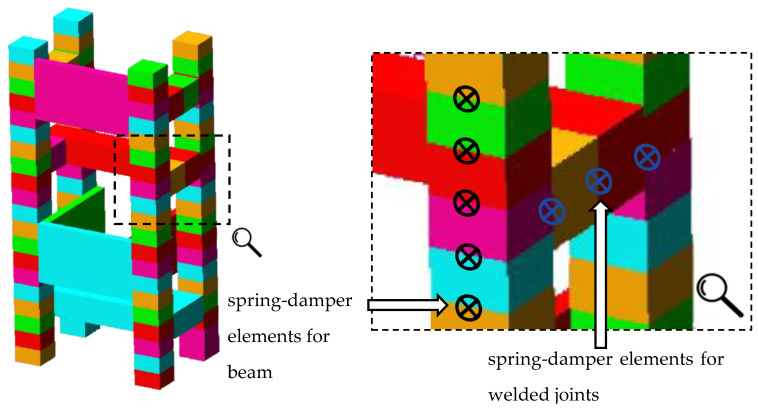
Rigid finite element model of the steel-polymer concrete frame.

Next, a rigid finite elements model for the steel-polymer concrete frame was built—Figure 6. The model consisted of 91 rigid finite elements; 76 spring-damping elements describing the stiffness and damping of the beam elements, defined analogously to the beam model; and 73 spring-damping elements, describing the stiffness and damping of welded joints between the individual beams. As a result, the model had 546 degrees of freedom.

### 3.3. Comparison of the Results of Model Calculations of the Basic Structural Component with the Results of the Experimental Tests

The results for the RigFEM and FEM models of the beam were compared to the experimental results. Table 5 compares the values of natural frequencies supplemented by the value of relative error defined as follows Equation (11):

(11) $$\delta =\left|\frac{f_{exp}-f_{model}}{f_{exp}}\right|\times 100\%$$

where $f_{exp}$—experimentally determined natural frequency; $f_{model}$—natural frequency determined for the rigid finite element model (RigFEM) and the finite element model (FEM).

**Figure 7:**
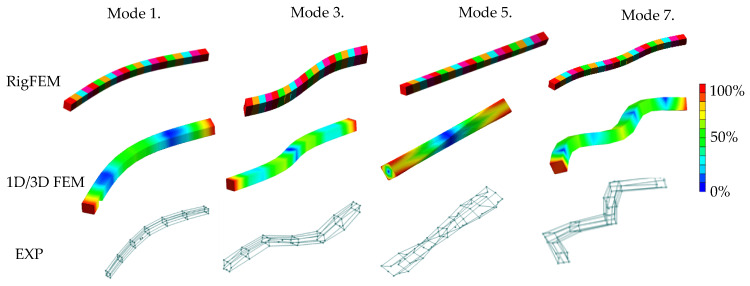
Selected mode shapes comparison for beam.

A comparison of the selected mode shapes determined by calculation and experimental studies is presented in Figure 7. Analyzing the presented comparison in Figure 7, one can see that a mode shape agreement in the analyzed frequency range was achieved, therefore it can be stated that a full structural compliance of the model was achieved.

**Figure 8:**
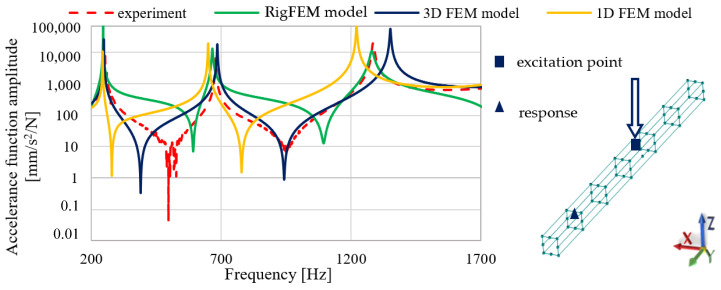
Frequency response function comparison for the 50 × 50 beam.

Figure 8 contains the frequency response functions comparison for the beam.

Analyzing the comparison of accelerance functions presented in Figure 8, it can be seen that a good agreement was achieved for all the models for the first two resonant frequencies both in terms of amplitude and the resonant frequencies values. For the third resonant frequency, the best fit is achieved by the RigFEM model; the 1D and 3D FEM models have a poorer accuracy in terms of the resonant frequency value and the corresponding accelerance amplitude.

**Figure 9:**
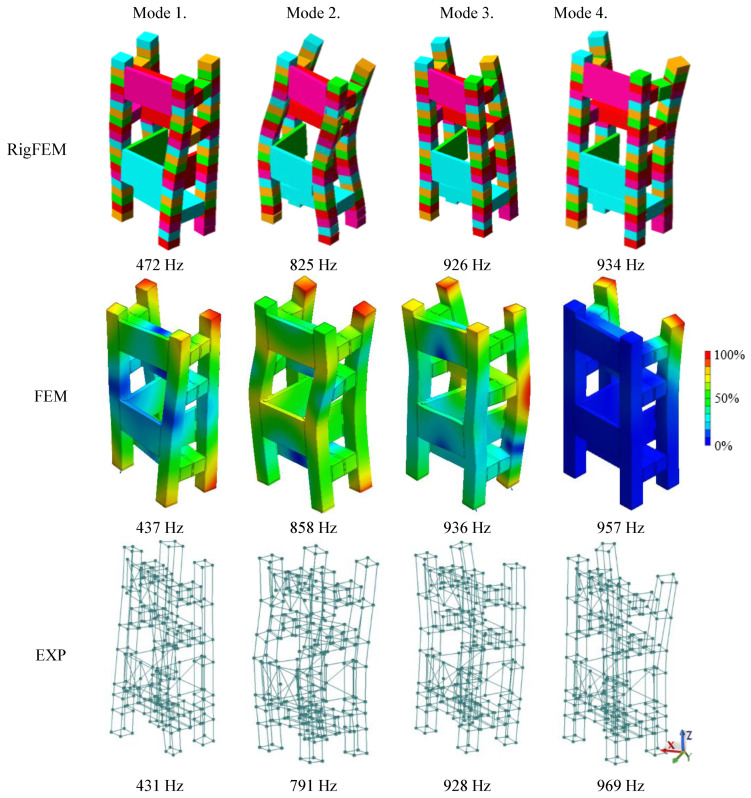
Comparison of the selected mode shapes between the RigFEM, FEM model, and the experimental results for the machine tool body.

Table 6 and Figure 9 compare the results of the calculations made for the RigFEM and FEM models of a steel-polymer concrete frame with the results of the experimental studies. 

Analyzing the comparison presented in Figure 9, it can be seen that, similar to the beam, a mode shape agreement for the frame was achieved, therefore it can be stated that a full structural compliance of the model was achieved.

**Figure 10:**
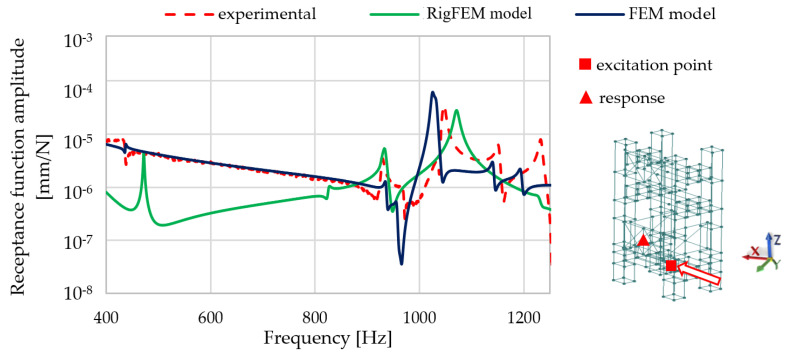
Selected receptance function comparison for the RigFEM, FEM model, and experimental data between the excitation point and the point where spindle is mounted in the X direction.

Figure 10 presents the FRF comparisons at selected points where the spindle is mounted.

Analyzing the comparison presented in Figure 10, it can be seen that the RigFEM model gives less accurate results compared with FEM model, in particular in the lower frequency range. For the RigFEM model, the amplitude mapping of the first resonance is inaccurate; a similar phenomenon can be seen for resonances close to the frequency of 1200 Hz.

## 4. Discussion

To summarize, it can be concluded that for the RigFEM model of the beam analyzed, the full structural compliance of the model—i.e., the compliance of the mode shapes—has been achieved in the frequency range under consideration. The relative error for the natural frequency values for the RigFEM model did not exceed 4%; on average it was 1.8%. Referring these results to those presented in [34,36], it can be seen that for the competitive FEM model, the relative error did not exceed 3%—on average, it was 1.8%. The 3D FEM did not exceed 4%—on average, it was 4.1% (1D FEM). For the continuous model based on Timoshenko beam theory, the relative error did not exceed 4%—on average, it was 3.1%. Additionally, for the Euler–Bernoulli beam model of a similar steel-polymer concrete beam (differing in a steel profile cross-section dimensions—70 × 70 mm) presented in [42], the relative error for the bending mode shapes did not exceed 13.2%—on average, it was 7.6%. Comparing the natural frequencies obtained on the basis of the RigFEM beam model with the models described in the cited works, it can be seen that th eRigFEM model has a slightly lower accuracy than the 3D FEM model and has a better accuracy than the 1D FEM model and the continuous models, especially the Euler–Bernoulli model. It should be also noted that for the RigFEM model, an acceptable compatibility of the frequency response functions for resonant frequencies has been achieved.

Comparing the RigFEM and FEM models, it can be seen that the model developed according to RigFEM is characterized by a 95% reduction in the number of degrees of freedom in the case of the 3D model, and 50% in the case of the 1D model. Reducing the number of degrees of freedom directly shortens the time needed for the calculation, without the need for the model order reduction presented in [43].

The differences between the RigFEM beam model and the experimental tests can result from the adopted simplifications—i.e., the use of a small number of rigid finite elements and spring-damper elements can adversely affect the accuracy of both natural frequency values and frequency response functions [29]. The adoption of an isotropic, homogenous, linear elastic model of material to represent heterogenous polymer concrete may also influence the accuracy of the model due to the possible uneven distribution of the stiffness and mass. However, it should be noted that these assumptions were made deliberately and with full awareness of their consequences and were intended primarily to reduce the dimensionality of the model. One way to improve the accuracy of the model may be to use a model updating algorithm.

It can be also concluded that, similarly to the beam, for the frame, a full structural compliance of the models was obtained, which was manifested by the compliance of the mode shapes. Comparing the natural frequency values for the RigFEM model, it can be seen that the relative error did not exceed 11%—on average, it was 5.5%. For the FEM model built according to methodology presented in [34], the relative error did not exceed 9%—on average, it was 2.4%. In addition, a satisfactory compliance of the frequency response functions was achieved for both the models.

Comparing the models developed in accordance with RigFEM and FEM, it can be seen that the model developed according to RigFEM is characterized by a 99.8% reduction in the number of degrees of freedom, directly influencing the time needed for the calculation.

The differences between the RigFEM frame model and the experimental data may result from similar reasons to the beam model. An additional source of inaccuracy may be the simplified method of modeling the welded joints, which was implemented using spring-damper elements connecting the centers of mass of the rigid end elements.

Therefore, it can be concluded that the RigFEM models developed for both a steel-polymer beam and a frame composed of such beams provide a reliable description of their dynamic properties. In addition, their small size makes them competitive in relation to the models developed in accordance with FEM.

## 5. Conclusions

The literature sources indicate that the finite element method is the most popular in engineering practice to anticipate the dynamic properties of steel-polymer concrete machine tool frames. However, using FEM models at an early design stage, when several structural variants need to be analyzed, can be time-consuming due to their high dimensionality.

Therefore, the presented rigid finite element methodology of modeling composite steel-polymer concrete structures can be a useful tool to assist engineers in a design decision-making process, owing to the possibility of developing reliable low-dimensional models.

The obtained results confirmed the initially formulated thesis that the rigid finite element method can be effectively used in modeling and calculating the dynamic properties (natural frequencies, mode shapes, and frequency response functions) of both simple steel-polymer beams and complex spatial concrete frames. The accuracy of predicting the dynamic properties of the steel-polymer concrete structures for the RigFEM model is only slightly inferior to the FEM model and is fully acceptable. On the other hand, using RigFEM models with a reduced dimensionality leads to the reduction of time necessary to perform the calculations, which may be beneficial in the case of multiple repetitions of the calculations for many different structural variants (when searching for the optimal solution, favor the RigFEM model over the FEM model).

The main limitation of the presented models is the frequency response function accuracy for the non-resonant frequencies. However, according to the fact that displacements for non-resonant frequencies are small in general, the accuracy of the presented method can be considered as satisfactory.

To summarize, the study presents a method of modeling steel-polymer concrete machine tool frames based on the rigid finite element method. Comparing the RigFEM models with the analytical and finite element ones, it can be stated that the developed method is an interesting alternative to them.

## Figures and Tables

**Table 1:** Composition of the applied polymer concrete filling (mass percentage).

Component	Epoxy Resin	Ash	Fine Fraction (0.25–2 mm)	Medium Fraction (2–10 mm)	Coarse Fraction (8–16 mm)
Percentage share of component weight	15%	1%	19%	15%	50%

**Table 2:** Material properties.

Parameter	Steel	Polymer Concrete
Young’s modulus E	210 ± 5 GPa	16.8 ± 0.2 GPa
Poisson’s ratio ν	0.28 ± 0.03	0.20 ± 0.05
Density ρ	7487 ± 35 kg/m^3^	2118 ± 6 kg/m^3^

**Table 3:** Parameters of signal acquisition.

Parameter	Value
Sampling rate	4096 Hz
Frequency resolution	0.5 Hz
Signal acquisition time	2 s
Frequency response function estimator	H1
Number of averages	10
Scaling of the frequency response function	global

**Table 4:** Loss factors for steel and steel-polymer concrete structure.

Parameter	Steel	Polymer Concrete
Loss factor η	0.00220 ± 0.00005	-
Equivalent loss factor	0.00480 ± 0.00024	

**Table 5:** Comparison of the natural frequencies between the rigid finite element model (RigFEM), 3D FEM, and 1D FEM and the experimental results for the beam analyzed.

Mode Number	Experimental Results	RigFEM Results	Relative Error δ_RigFEM_	3D FEM Results	Relative Error δ3_D-FEM_	1D FEM Results	Relative Error δ_1D-FEM_
1	247 Hz	244 Hz	~1%	248 Hz	<1%	243 Hz	<2%
2	251 Hz	245 Hz	~2%	248 Hz	~1%	243 Hz	<2%
3	676 Hz	664 Hz	~2%	685 Hz	~1%	650 Hz	~4%
4	678 Hz	665 Hz	~2%	685Hz	~1%	650 Hz	<4%
5	1276 Hz	1224 Hz	~4%	1241 Hz	<3%	1184 Hz	~7%
6	1282 Hz	1275 Hz	<1%	1326 Hz	~3%	1223 Hz	<5
7	1282 Hz	1275 Hz	<1%	1326 Hz	~3%	1223 Hz	<5

**Table 6:** Comparison of the natural frequencies between the RigFEM and FEM models and the experimental results of a machine tool body composed of 50 × 50 mm steel beams filled with polymer concrete.

Mode Number	Experimental Results	RigFEM Results	Relative Error δ_RigFEM_	FEM Results	Relative Error δ_FEM_
1	431 Hz	472 Hz	~9%	437 Hz	<1%
2	791 Hz	825 Hz	~4%	858 Hz	<9%
3	928 Hz	926 Hz	<1%	936 Hz	<1%
4	969 Hz	934 HZ	~4%	957 Hz	~1%
5	1041 Hz	1070 Hz	<3%	1032 Hz	<1%
6	1151 Hz	1230 Hz	<7%	1141 Hz	<1%
7	1232 Hz	1372 Hz	~11%	1195 Hz	~3%
